# Interpersonal synchronization of spontaneously generated body movements

**DOI:** 10.1016/j.isci.2023.106104

**Published:** 2023-02-01

**Authors:** Atesh Koul, Davide Ahmar, Gian Domenico Iannetti, Giacomo Novembre

**Affiliations:** 1 Neuroscience of Perception and Action Lab, Italian Institute of Technology (IIT), Viale Regina Elena 291, 00161 Rome, Italy; 2 Neuroscience and Behaviour Lab, Italian Institute of Technology (IIT), Viale Regina Elena 291, 00161 Rome, Italy

**Keywords:** Health sciences, Body system

## Abstract

Interpersonal movement synchrony (IMS) is central to social behavior in several species. In humans, IMS is typically studied using structured tasks requiring participants to produce specific body movements. Instead, spontaneously generated (i.e., not instructed) movements have received less attention. To test whether spontaneous movements synchronize interpersonally, we recorded full-body kinematics from dyads of participants who were only asked to sit face-to-face and to look at each other. We manipulated interpersonal (i) visual contact and (ii) spatial proximity. We found that spontaneous movements synchronized across participants only when they could see each other and regardless of interpersonal spatial proximity. This synchronization emerged very rapidly and did not selectively entail homologous body parts (as in mimicry); rather, the synchrony generalized to nearly all possible combinations of body parts. Hence, spontaneous behavior alone can lead to IMS. More generally, our results highlight that IMS can be studied under natural and unconstrained conditions.

## Highlights

• Spontaneous body movements synchronize interpersonally
• Synchrony only requires visual contact (not a structured social task)
• Synchrony emerges rapidly and entails nearly all body part combinations
• Spontaneous behavior alone can lead to interpersonal synchrony

## Graphical abstract

**Figure:**
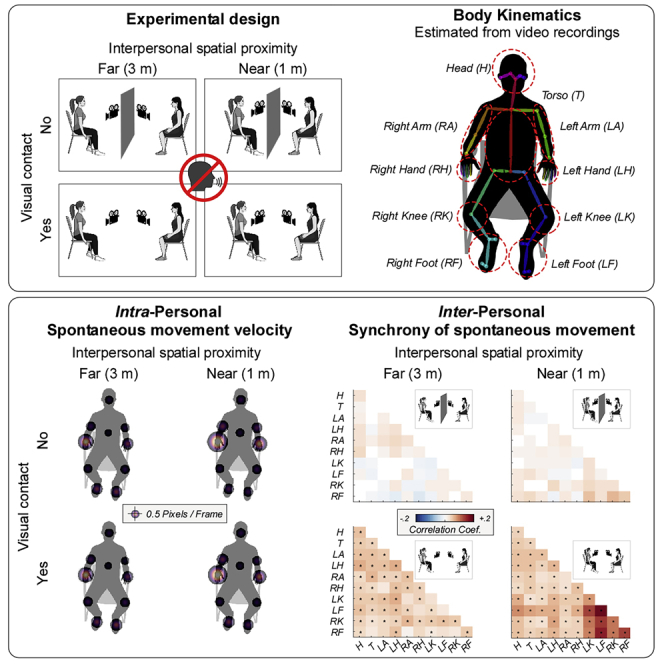

## Introduction

Interpersonal movement synchrony (IMS)—the temporal alignment of one’s movements with those of others—is an important aspect of many social interactions. In nonhuman species, it is often used as a means of cooperation and communication.^1^^,^^2^ In humans, IMS has been suggested to have important prosocial consequences.^3^^,^^4^^,^^5^ For instance, individuals who synchronize their walking pace are more likely to cooperate with each other.^6^ Likewise, the degree to which two individuals synchronize in a finger-tapping task predicts their subsequent feeling of affiliation.^7^

IMS is typically studied in the context of structured tasks where participants are explicitly asked to produce specific body movements (e.g.,^7^^,^^8^^,^^9^^,^^10^^,^^11^^,^^12^^,^^13^^,^^14^). Under these conditions, IMS not only emerges intentionally, as when soldiers march in unison, but also unintentionally, as when two strangers happen to synchronize the pace of their footsteps.^12^^,^^13^^,^^15^^,^^16^^,^^17^^,^^18^ Indeed, multiple studies have characterized the automatic emergence of IMS when participants interact with each other and, notably, perform a task together. For example, Schmidt and O’Brien demonstrated that pairs of participants swinging handheld pendulums to their preferred frequency automatically align their relative phase angles (at 0° or 180° degrees), even if they are not instructed to do so.^14^ Notably, such unintentional IMS displays unstable and non-steady-state characteristics that are typical of self-organized, coupled oscillators showing attraction to certain regions of the phase space (0° and 180° in the case described above).^13^^,^^18^^,^^19^^,^^20^^,^^21^

While IMS has, so far, been exclusively examined when participants are instructed to perform movements, spontaneous movements have received less attention. This is surprising considering that interacting individuals not only produce movements that are technically necessary to fulfill a given task but also exhibit *spontaneous* (i.e., not instructed) movements that can serve communicative functions.^22^^,^^23^^,^^24^ For example, while playing music, musicians exhibit ancillary movements (e.g., body sway and head nodding) that are not strictly essential for the musical output but might help co-performers to synchronize^25^^,^^26^^,^^27^ or a student to learn new musical material.^24^^,^^28^ Similarly, addressees in a conversation spontaneously produce long eye blinks that can be read as a signal regulating turn-taking behavior.^23^^,^^29^ Notably, as compared to instructed movements, spontaneously produced movements rely on distinct cognitive and neurobiological resources^22^^,^^30^^,^^31^^,^^32^^,^^33^^,^^34^ and therefore their interpersonal synchronization should not be taken for granted.

**Figure 1:**
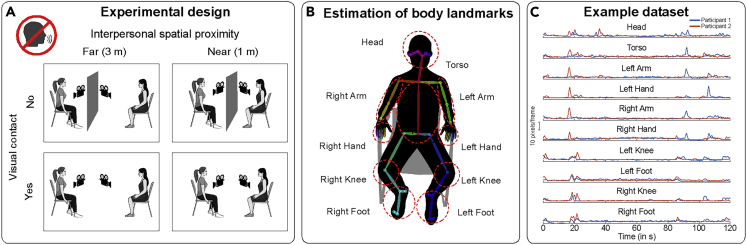
Experimental setup and dependent variable (A) Experimental design. Twenty-three pairs of participants were seated facing each other while we video-recorded them. The video recordings were obtained from two cameras placed laterally to the participants. We manipulated visual contact (Vision, No Vision) and interpersonal spatial proximity (3 meters, 1 meter) in a 2x2 factorial manner. During the No Vision conditions, a screen was placed in between the two participants to prevent them from seeing each other. Throughout the experiment, the participants were asked to relax, behave naturally and, when not prevented from the screen, look at the other person (without speaking or making co-verbal gestures). (B) Estimation of the body landmarks. We used the video recordings to predict 67 body landmarks for each participant [25 landmarks on different body parts (across the head, torso, legs, and feet) and 21 landmarks per hand]. This prediction was based on an automated machine learning estimation (i.e., OpenPose^35^). The body landmarks were then grouped to represent 10 different body parts (highlighted with red dashed ellipses). (C) Representative time series indexing movement velocity (pixels/frame) from two participants forming a dyad. Note that spontaneous movements are often contingent across the two participants (note the overlapping data points).

To directly test whether spontaneous movements synchronize interpersonally, we designed a task-free interaction study in which 23 dyads of participants were only asked to sit face-to-face and to look at each other (Figure 1A), without speaking or making co-verbal gestures. Exploiting the absence of a structured social interaction, this paradigm gave us the chance to record spontaneous movements alone—without confounding them with any task-related movement.

Body-part-specific kinematics (specifically, movement velocities) were automatically extracted from video recordings using OpenPose, a human pose detection library relying on machine learning.^35^ As an open-source and validated method, OpenPose achieves high accuracy in automatically extracting movement kinematics of multiple body parts (including head, torso, arms, hands, legs, and feet) from video images (e.g.,^36^^,^^37^). This approach is preferable to previous video-based methods that either rely on raters manually labeling videos in a frame-by-frame manner (e.g.,^38^^,^^39^^,^^40^^,^^41^), or image analysis techniques such as motion energy (e.g.,^42^^,^^43^^,^^44^). Indeed, these more traditional approaches fall short in providing highly accurate kinematic information because they are (i) prone to human error, (ii) rater dependent, (iii) generally very time-consuming, and (iv) rarely comprehensive in characterizing full-body kinematics. Furthermore, even though kinematic estimates based on video cameras are not as precise as those yielded by infrared cameras [(e.g., systems such as Vicon, Optitrack, and Qualisys, see^9^^,^^42^^,^^45^^,^^46^^,^^47^^,^^48^, the former are less expensive, less cumbersome, and they do not involve wearing specialized markers, making this method very accessible and suited for ecological and field studies.^49^^,^^50^

We investigated whether (i) IMS might emerge from spontaneous behavior in the absence of a structured task and, if so, (ii) which specific body parts would move synchronously across participants and, (iii) how long information transfer might take. We accomplished this by manipulating two factors that implicitly influence social behavior: visual contact and interpersonal spatial proximity (Figure 1A). For the former manipulation (visual contact), participants were either able (Vision) or not able (No Vision) to see each other. For the latter (interpersonal spatial proximity), they were either seated 1 meter (Near) or 3 meters (Far) apart from each other. We expected the emergence of IMS only when participants could see each other. We also explored whether IMS would change as a function of interpersonal spatial proximity.

## Results

We analyzed the spontaneous body movement of 10 representative body parts (as illustrated in Figure 1B). Our analysis focused on “movement velocity”, indexing body part displacement over time irrespective of the spatial direction of the movement (similar to computing movement speed; see Figure 1C for representative time series, and the STAR Methods for a detailed description of the pipeline). We first examined whether movement velocities differed across conditions at an individual (intra-personal) level. We then tested whether movement velocities synchronized interpersonally. Finally, we attempted to estimate how rapidly IMS emerged.

### Spontaneous (intra-personal) movement velocity

**Figure 2:**
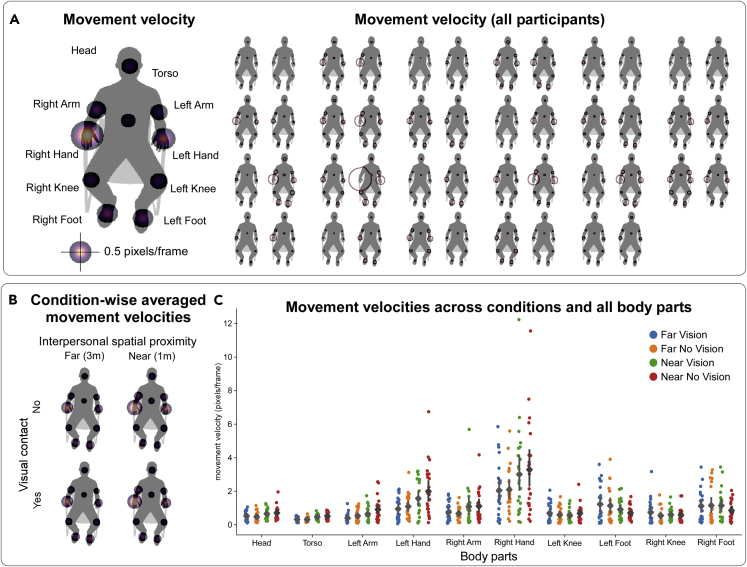
Spontaneous (intra-personal) body movement velocity (A) Movement velocity averaged across conditions (left: group average; right: all participants). These data show that the hands and the feet displayed the highest range of velocities, while the head and the torso displayed the lowest. (B) Averaged movement velocities are depicted separately for each condition. These data show that body movement velocity was comparable across Vision and No Vision conditions, but higher when participants were relatively closer to each other. (C) Same as (B), displaying data for each dyad (dots). The mean for each condition is represented by a diamond while the vertical lines represent 95% confidence intervals.

To understand if individual participants moved their body at different velocities across our experimental conditions, we estimated the range of their spontaneous movement velocities. Overall, the body parts that showed the highest range of movement velocity were the hands (Right Hand: 2.61 pixels/frame; Left Hand: 1.40 pixels/frame) and the feet (Left Foot: 1.05 pixels/frame; Right Foot: 0.99 pixels/frame), while the Head (0.59 pixels/frame) and the Torso (0.40 pixels/frame) were associated with the lowest velocity range (Figure 2A). An ANOVA on these data yielded a main effect of “body part” (*F*_1.57,34.58 -_ 26.82; p < 0.001; generalized *η*^*2*^ = 0.30) (Figure 2B), which was driven by significantly higher movement velocities associated with both Right Hand and Left Hand as compared to Head and Torso (p < 0.001 Holm–Bonferroni corrected). Because the hands can move faster than other body parts, simply due to their biomechanical properties, this result is to be expected.

Individual movement velocity did not change depending on whether participants could see each other. In line with this, the ANOVA yielded a non-significant main effect of “visual contact” (*F*_1,22_ = 0.29; p = 0.6; generalized *η*^*2*^ = 0.0). Furthermore, “visual contact” did not interact with any of the other variables, as indicated by a non-significant interaction between “visual contact” and “interpersonal spatial proximity” (*F*_1,22_ = 0.68; p = 0.42; generalized *η*^*2*^ = 0.001), between “visual contact” and “body part” (*F*_3.61,79.31_ = 1.94; p = 0.12; generalized *η*^*2*^ = 0.006), or between “visual contact”, “interpersonal spatial proximity”, and “body part” interaction (*F*_3.08,67.65_ = 0.89; p = 0.46; generalized *η*^*2*^ = 0.002).

Interestingly, movement velocity changed as a function of interpersonal spatial proximity: dyads moved faster when they were relatively closer to each other (Figure 2C). This result was supported by a significant main effect of “interpersonal spatial proximity” (*F*_1,22 -_ 15.95; p < 0.001; generalized *η*^*2*^ = 0.016) (Figure 2B), and also by an interaction between “interpersonal spatial proximity” and “body part” (*F*_1.74,38.26 -_ 7.33; p = 0.003; generalized *η*^*2*^ = 0.047). The latter interaction further indicated that this proximity-driven increase of movement velocity was most pronounced for body parts such as the Right Hand (Figure 2C).

### Interpersonal synchrony of spontaneous movements

**Figure 3:**
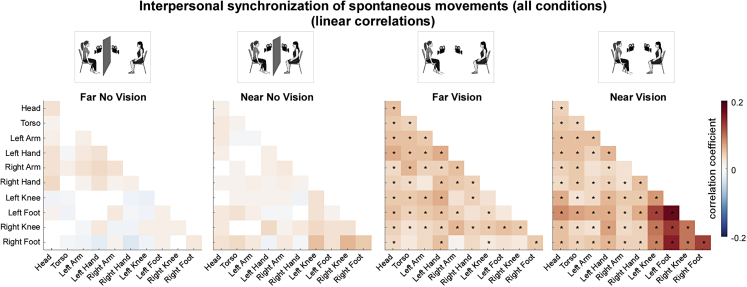
Interpersonal synchrony of spontaneous movements (all conditions - linear correlations) Averaged (Pearson’s) correlation coefficients indexing interpersonal movement synchrony, across all body part combinations, separately for each experimental condition. Non-parametric permutation-based statistical tests revealed that spontaneous movements synchronized across multiple body parts, only when participants could see each other (∗p < 0.05 FDR-corrected). See also Figures S1 and S3.

We quantified IMS by correlating (using Pearson’s correlations) the movement velocity time series of the two participants forming a dyad. Spontaneous movement velocities synchronized across participants only when they could see each other (i.e., Far Vision and Near Vision conditions). Notably, IMS entailed nearly all body part combinations (Figure 3). This result was confirmed by permutation-based statistical tests [FDR-corrected p values <0.05; *Cohen’s* *d* ranging from 0.4 (Head – Right Arm) to 0.86 (Left Foot – Right Knee) for Far Vision; and 0.36 (Left Knee – Torso) to 1.33 (Left Foot – Right Foot) for Near Vision].

**Figure 4:**
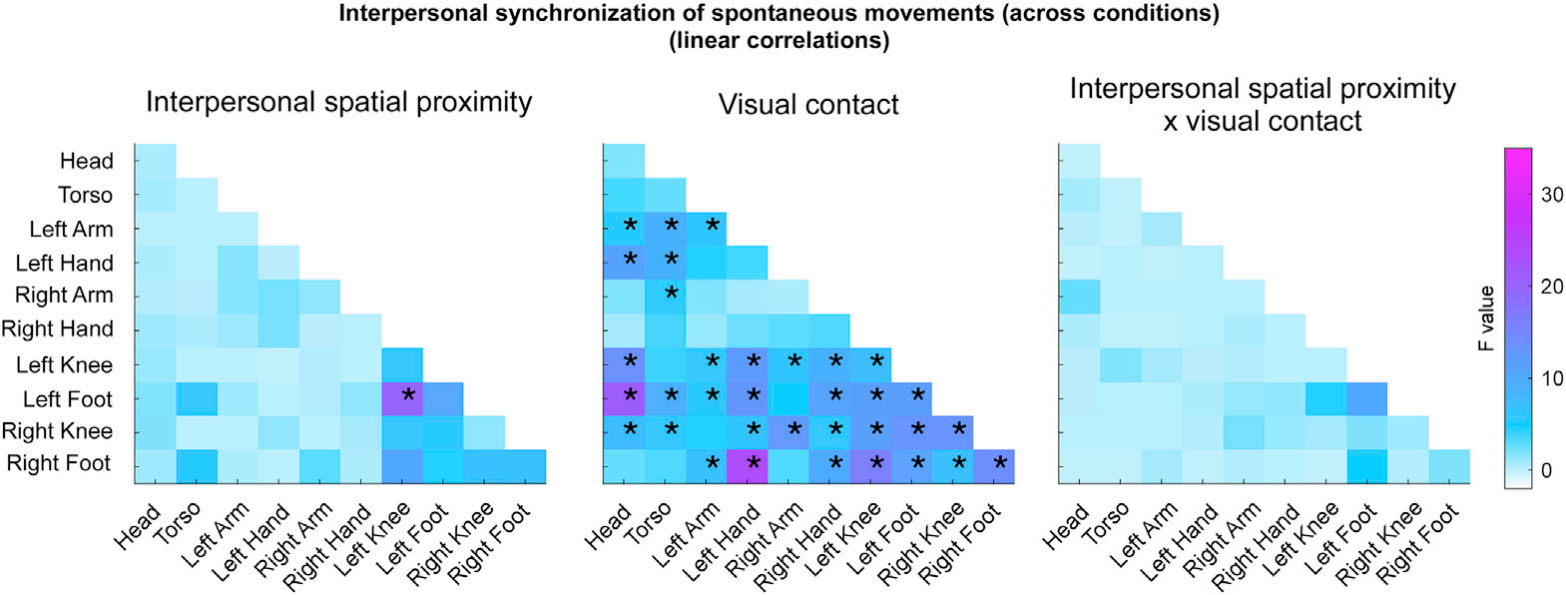
Effects of interpersonal spatial proximity and visual contact on interpersonal synchrony of spontaneous movements (across conditions - linear correlations) F-values resulting from non-parametric ANOVAs comparing all experimental conditions (separately for each body part combination). The ANOVAs yielded several significant main effects of “visual contact” on interpersonal movement synchrony, entailing most body part combinations (as well as a single main effect of “interpersonal spatial proximity”, selectively entailing the Left Foot and the Left Knee). The interaction between the “interpersonal spatial proximity” and “visual contact” was not statistically significant (∗p < 0.05 FDR-corrected). See also Figures S2 and S4.

Having established that IMS only emerged in the Vision conditions (Figure 3), we also compared all conditions using a 2x2 ANOVA analysis with “visual contact” and “interpersonal spatial proximity” as factors (Figure 4). Consistently with the previous analysis, the ANOVA yielded a main effect of “visual contact”, again indicating that IMS emerged only when participants could see each other (FDR-corrected p values <0.05; generalized *η*^*2*^ ranging from 0.045 to 0.22). Notably, IMS appeared to be mostly associated with movements of the upper and lower limbs (Figure 4).

There was no considerable evidence for an effect of “interpersonal spatial proximity” on IMS [i.e., only one body part combination (Left Foot – Left Knee) yielded a significant result; FDR-corrected p < 0.05; generalized *η*^*2*^ = 0.17; Figure 4]. Furthermore, even though IMS appeared to be numerically higher during the Near Vision (average correlation coefficients ranging between 0.0270 and 0.1778) compared to Far Vision condition (average correlation coefficients ranging between 0.0231 and 0.0649), this was not substantiated by a significant interaction (compare Figures 3 and 4).

We also conducted a series of control analyses. First, we confirmed the above results (obtained using a permutation-based non-parametric analysis) using a traditional parametric analysis (see Figures S1 and S2). Second, we performed an additional analysis based on a qualitative assessment of participants’ behavior [i.e., relying on a human rater visually inspecting all videos, in a frame-by-frame fashion, and labeling all perceivable instances of movement (see Figure S3)]. This additional analysis also corroborated the above results. Finally, because the above results were all based on linear correlations, and in order to make our results comparable to others in the field,^13^^,^^14^^,^^17^ we explored whether IMS modulations were also observable using circular measures. For this purpose, we estimated the instantaneous phase of the two movement time series (associated with the two participants forming each dyad), and then estimated the circular correlation and the circular variance of the continuous relative phase. The results of these analyses were also consistent with those reported above (see Figure S4).

### Rapid emergence of interpersonal synchrony

**Figure 5:**
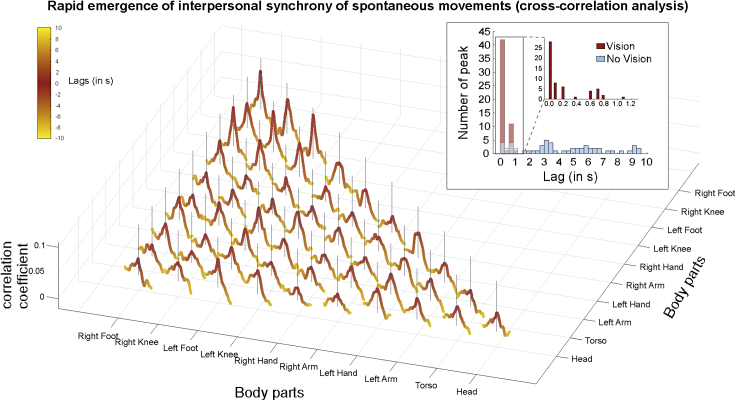
Rapid emergence of interpersonal synchrony of spontaneous movements We cross-correlated participants’ movement velocity time series (separately for each body part combination) to estimate the lag at which the highest coefficient (i.e., the peak) occurred. The lags associated with the highest coefficients were notably close to lag “0”, implying that IMS emerged very quickly, and this was so across all body part combinations. Each colored line represents correlation coefficients—with ±10 s time lags—averaged across Vision trials and conditions. The vertical black line represents the lag “0” and its height is proportional to the y axis (indexing the correlation coefficient). The distribution of the time lags associated with the peaking coefficients is shown in the inset (all body part combinations are pooled here). For a comparison, the distribution associated with the No Vision conditions is also shown. Note that the peaks associated with the Vision conditions have remarkably shorter lag compared to those associated with the No Vision conditions (Wilcoxon signed rank test Z = 6.23, p < 0.001).

Finally, focusing on the Vision conditions, we cross-correlated participants’ movement velocity time series to shed light upon the timing of information transfer, i.e., how long a movement performed by one participant would take to possibly elicit another movement in the partner. The results from this analysis indicated that the cross-correlation coefficients (indexing similarity between time series of the two partners as a function of their relative temporal displacement) peaked (on average) very close to lag “0”. Specifically, the averaged lags of the correlation peaks were as short as 200 ms, with the first quartile falling on 0 ms and the third quartile falling on 200 ms (Figure 5). Notably, 78.20% of the peaks had lags shorter than 500 ms (see inset in Figure 5). Together, these results indicate that information transfer was remarkably fast, and that IMS emerged very quickly, i.e., within few hundreds of milliseconds.

## Discussion

We investigated whether spontaneous movements, produced during a task-free interaction, synchronize interpersonally within dyads of participants. We estimated the velocity of spontaneous body movements from video recordings, using an automated machine learning approach, and examined how these velocities changed as a function of visual contact and interpersonal spatial proximity, both intra- and interpersonally. We report that visual contact (but not interpersonal spatial proximity) was sufficient to trigger the emergence of IMS. Such synchrony (i) emerged very rapidly (within few hundreds of milliseconds), (ii) affected nearly all body parts, and (iii) was most pronounced in lower and higher limbs. In contrast, individual movement velocity did not change as a function of visual contact but was enhanced when participants were closer to each other. These results will now be discussed in this order.

### Synchronization of spontaneous movements depends on visual contact

We demonstrated that spontaneously generated movements synchronize across participants, but only when they can see each other. We observed higher synchrony across hands and feet, as opposed to other body parts such as the torso or the head. This might be because participants were sitting on a chair and therefore were not easily able to move body parts such as the torso. A different experimental scenario, e.g., having participants standing instead of sitting, might possibly lead to stronger synchronization of other body parts.

It is important to distinguish the observed IMS from previously described mimicry-like phenomena, i.e., the general tendency of one individual in a dyad to imitate the partner’s body posture, movement, or facial expression.^51^^,^^52^^,^^53^^,^^54^ For instance, participants have been shown to mimic the mannerisms and facial expressions of a confederate partner (e.g., participants rubbed their face more frequently when the confederates rubbed their face^51^^,^^55^) or match their body postures during a conversation (e.g., greater recurrence of hip displacements between two participants engaged in conversation^54^^,^^56^^,^^57^). It should be noted that these previously characterized phenomena (i) selectively entail homologous body parts and/or (ii) do not necessarily imply interpersonally synchronized actions. That is, these phenomena imply reproducing the behavior of another individual using homologous body parts and often following relatively large time lags (in the second timescale). Instead, in the current study, we observed that IMS (i) emerged very quickly (within few hundreds of milliseconds, see Figure 5) and, notably, (ii) was not solely restricted to homologous body parts but entailed nearly all body part combinations. Hence, our results cannot be interpreted as the manifestation of a mimicry-like phenomenon.

We suggest that the IMS we observed derives from fast and unintentional physiological responses to partners’ body movements, which could have been processed as salient stimuli, i.e., sudden stimuli that are likely to catch an observer’s attention.^58^^,^^59^^,^^60^^,^^61^^,^^62^ Indeed, the body movements produced by our participants followed a sharp and fast-rising profile that could act as a synchronizing signal (for an example, see Figure 1C). Notably, previous neurophysiological studies have demonstrated that salient stimuli with a fast-rising profile trigger fast behavioral responses,^63^^,^^64^^,^^65^^,^^66^^,^^67^^,^^68^ which could potentially facilitate the emergence of IMS. Consistently with these observations, Wass and colleagues recently proposed that, during an interaction, sharp changes (or “edges”) in behavioral responses (such as the onset of gaze or of a vocalization) could act as phase-resetting cues that could lead to behavioral and neural alignment.^69^

While IMS relied strongly on visual contact, we found negligible or no effect of interpersonal spatial proximity on IMS (i.e., only 1 out of 55 body part combinations was marginally modulated by interpersonal spatial proximity). Furthermore, even though the effect of visual contact on IMS appeared to be stronger when participants were closer, we found no evidence of a significant interaction. Provided that a relationship between interpersonal spatial proximity and social behavior is to be expected given previous research,^70^^,^^71^^,^^72^^,^^73^^,^^74^^,^^75^^,^^76^^,^^77^^,^^78^^,^^79^ we might not have observed it because of two possible reasons. Either the effect of interpersonal spatial proximity was mitigated by other changing variables that we did not measure such as, for instance, attentional focus (see e.g.,^76^^,^^80^^,^^81^^,^^82^), or we might conclude that such relationship does not necessarily manifest in terms of IMS. If the latter, proximity might trigger distinct prosocial mechanisms, other than the ones leading to IMS. In line with this notion, while Lahnakoski and colleagues found modulations of both interpersonal spatial proximity and IMS during natural conversation and joint action tasks, their effects were independent, i.e., only interpersonal spatial proximity correlated with the quality of social interaction.^72^ Hence, it is possible that interpersonal spatial proximity might not exert a direct influence on IMS, but may have other interpersonal consequences (e.g., on interpersonal rapport), possibly mediated by distinct mechanisms (as discussed in the next section).

### Movement velocities depend on interpersonal spatial proximity but not on visual contact

Besides examining IMS, we also probed the range of spontaneous movement velocities produced by the participants at the intrapersonal level. We found that hands and feet showed the highest velocities. This is in agreement with biomechanical principles, according to which hands and feet can move faster than other body parts such as the head or torso.^83^

Movement velocities of the participants were not influenced by visual contact, but depended on the interpersonal spatial proximity and on the specific body part. These results have two main implications. First, they suggest that participants made similar amounts of movements whether they could see each other or not, i.e., they did not generally increase (or decrease) their body movements simply because they could see a partner. This rules out the possibility that the synchrony in Vision conditions is a mere consequence of participants moving less or more when seeing each other.

Second, our results highlight that the range of spontaneous movements increases when participants are close to each other, irrespective of the visual contact. Somewhat similar results have been observed in a previous study where two participants were asked to walk face-to-face (one forwards and the other backwards) while maintaining a constant distance between them: participants moved their heads at a higher velocity when they were asked to walk 1 m apart compared to when they were asked to walk 3 m apart.^84^ Our results confirm this finding, while examining spontaneous movements, and further add to the literature by providing evidence that the range of movement velocity increases even when the participants are prevented from observing each other. It is possible that this result reflects an increase in the potential for action and interaction when another individual is closer.^85^ This would be in agreement with the recent reconceptualization of peripersonal space as a set of continuous fields according to which the brain responds to environmental events based on their behavioral relevance for actual or potential actions, rather than simply based on distance from the body.^86^^,^^87^^,^^88^^,^^89^ Future studies could further clarify this point.

### Implications for future studies

The fact that spontaneous movements can synchronize interpersonally has two important implications. First, it indicates that the mechanisms underlying IMS are quite powerful, capable of triggering/inhibiting actions (in an observer) ex novo, not simply modulating the timing of ongoing actions (as previously observed, see^8^^,^^90^ for a review). Indeed, even though our participants were not required to move, they did so in response to their partners’ movements. This finding is consistent with dynamical systems theories predicting that synchronization arises from coupled, self-organizing processes.^91^^,^^92^^,^^93^ Our study specifically demonstrated that spontaneous dyadic movements couple through mere visual contact and without the need for any external instruction. Future studies might also investigate how such synchronization of spontaneous movements emerges specifically, e.g., taking into account individual movement frequencies, the field of view, or attentional mechanisms—all factors that should impact upon the establishment and maintenance of IMS.^12^^,^^13^^,^^80^^,^^81^^,^^94^^,^^95^^,^^96^

Second, our findings have implications for previous studies that have investigated interpersonal synchrony while participants performed a task together (as reviewed in the study by ^8^^,^^30^^,^^90^). It is possible that participants in these experiments also produced spontaneous (i.e., task-unrelated) synchronized behaviors that might have influenced the performance of the task itself (e.g., in the study by ^72^^,^^97^). Indeed, spontaneously arising behaviors are readily exhibited by most animals and represent a prerequisite for understanding behavioral dynamics in complex settings.^98^^,^^99^ Recently, many researchers are recognizing the significance of studying spontaneous, uninstructed behavior.^72^^,^^78^^,^^97^^,^^100^^,^^101^^,^^102^^,^^103^^,^^104^ Such behaviors, often mislabeled as noise, are being increasingly recognized as non-random phenomena that have a strong bearing on the interaction itself as well as on the performance of subsequent tasks.^98^ For instance, one recent study suggested that spontaneous movements better predict the quality of social interaction and individual traits as compared to movements performed according to task instructions.^72^ Together, these and our findings highlight how it is not only feasible but also necessary to pay more attention to spontaneous movements and their role in social behavior.

### Limitations of the study

The current study demonstrated that spontaneous body movements can lead to IMS. This was shown specifically in dyads of participants familiar with each other (see STAR Methods). Previous studies using structured tasks have shown that interpersonal familiarity can indeed boost the strength of IMS.^53^^,^^105^^,^^106^^,^^107^^,^^108^^,^^109^ Whether familiarity is a prerequisite for spontaneous movements to synchronize remains an open question for future research.

## STAR★Methods

### Key resources table

**Table:** 

REAGENT or RESOURCE	SOURCE	IDENTIFIER
**Deposited data**		

Kinematics data	This paper	https://doi.org/10.48557/4K3WZI
Custom analysis code	This paper	https://github.com/ateshkoul/interpersonal_movement_synch/

**Software and algorithms**		

synchCams	Atesh Koul; https://pypi.org/project/synchCams/	RRID:SCR_023152
OpenPose	Cao et al., 2017^35^	https://github.com/CMU-Perceptual-Computing-Lab/openpose
MATLAB	MathWorks; http://www.mathworks.com/products/matlab/	RRID: SCR_001622
Circular statistics Toolbox	Philipp Berens; https://www.mathworks.com/matlabcentral/fileexchange/10676-circular-statistics-toolbox-directional-statistics	RRID:SCR_016651

### Resource availability

#### Lead contact

Further information and requests for resources should be directed to and will be fulfilled by the lead contact, Atesh Koul (atesh.koul@iit.it).

#### Materials availability

This study did not generate new unique reagents or materials.

### Experimental model and subject details

#### Participants

A total of forty-six individuals (26 females; mean age 21.43 years, range 18–30 years) formed twenty-three dyads [13 same sex (8 female-female and 5 male-male) and 10 different sex dyads]. All pairs of participants were familiar with each other (years of familiarity with the partner were 6.59 ± 5.08 SD years; partner subjective closeness was rated 7.87 ± 2.27 SD on a scale of 1–10 where 1 is the lowest and 10 is the highest subjective closeness). Most of the participants were right-handed (i.e., 38/46 participants; >82% of the sample). All participants had normal or corrected-to-normal vision and no history of psychological or neurological disorders. The participants were naïve to the hypotheses of the study and were reimbursed with €25 for their participation in the experiment. Informed consent was obtained for each participant prior to the start of the experiment and all procedures were carried out in accordance with the local ethical committee and the revised Helsinki Declaration (World Medical Association, 2008).

### Method details

#### Experimental design and procedure

The current study analyzes part of the experimental data collected in a previous study (Koul et al., under review). Briefly, the entire experiment comprised four main experimental conditions that were organized according to a 2x2 factorial design (Figure 1A). We manipulated visual contact, i.e., whether the two participants forming the dyad could see each other or not (Vision, No Vision), and interpersonal spatial proximity, i.e., whether the participants forming the dyad were seated either 3 meters or 1 meter apart from each other (Far, Near).

We collected the data over repetitions (trials), each of which lasted 120 seconds (i.e., 2 minutes). We collected 3 trials per condition, for a total of 12 trials. The trials were further grouped into 3 blocks, each including 4 trials (1 per condition). The order of the trials was randomized (with one caveat: the two Far and the two Near conditions were always subsequent to one another in order to minimize the physical displacement of the participants throughout the experimental procedure). In the current manuscript, we focus on these four core conditions (notably, the original study included additional control conditions that did not entail video recordings and therefore, could not be of use here).

Before the start of the experiment, participants were provided with information about the equipment used to collect the data. Throughout the experiment, participants were asked to simply relax and act spontaneously while looking at each other (unless prevented by a screen that was placed in between the two participants; see also Figure 1A). Specifically, participants were asked to relax, behave naturally and when possible, look at the other person (not necessarily making eye contact) [This is the original text that the experimenter read to the participants before initiating the task (in Italian): ”*Il vostro compito è semplice: restare rilassati sulla sedia, comportarsi in modo naturale e, quando è possibile, guardare l’altra persona (non necessariamente negli occhi).”*]*.* Participants were not permitted to communicate verbally or through co-verbal gestures. We further specified that participants were not required to necessarily look at each other’s faces or eyes, but rather they were generally asked to look at the body of their partner. Following the end of the experiment, participants were informed about the scope of the study.

#### Behavioral recording

Video recordings were used to quantify the spontaneous body movements produced by participants. We recorded body movements from both participants of a dyad simultaneously using a dual video camera setup of two standalone cameras (SVPRO USB Webcam 5-50mm Varifocal Lens) mounted on tripod stands.

The cameras framed the two participants from the front side, having a slightly tilted (∼30°–45° with respect to the participants) aerial- and side-view (Figure 1A). The distance between the cameras and the participants changed minimally across Near (160 and 180 cm) and Far (316 and 260 cm) conditions. A custom python library ‘synchCams’ (https://pypi.org/project/synchCams/) orchestrated the simultaneous acquisition of videos from the two participants. This library allows a frame-locked dual video recording (i.e., the system acquired frames from the two cameras in an alternating fashion). ‘synchCams’ utilizes the python-based libraries - ‘opencv’ (https://opencv.org/) for video capture, ‘pyserial’ (https://pythonhosted.org/pyserial/) for access to the serial port, and ‘socket’ (https://docs.python.org/3/library/socket.html) for communication over ethernet.

In addition to these video recordings, we also captured videos from a binocular, lightweight eye-tracking system [Pupil Labs Core; Pupil Labs, Berlin, Germany^110^]. The eye-tracking system consisted of three different cameras. Two IR spectrum cameras monitored the two eyes of the participants simultaneously (120 Hz sampling frequency; 320 × 280 pixels). A third (head-mounted) camera was mounted on the forehead of the participant and recorded videos from the participant’s viewpoint (100° fisheye field of view; 30 Hz sampling frequency; 1,280 × 720 pixels). Data were sampled using the pupil capture software (Pupil Labs, Berlin, Germany; version 1.23). Note that these videos could only be used when recording data from the Vision conditions, i.e., when the participants could see each other because there was no screen blocking their view of the partner. Therefore, these videos could only be used for (i) a qualitative assessment made by a human rater (aimed at confirming the results of the automatized pipeline) and (ii) evaluating compliance with the experimental instructions, as described in the section below.

#### Evaluating compliance with experimental instructions

To confirm that the participants followed the instructions and actually looked at each other, we analyzed the eye-gaze behavior of each participant using data retrieved from the eye trackers. We estimated how often the participants looked at the partner’s (i) body and (ii) face. The former was performed by first estimating the area occupied by the partner’s body on each image, something we achieved using a pre-trained DeepLabV3 model with a ResNet-101 backbone.^111^ Next, this information was combined with the gaze information resulting in a binary code (1 if gaze location overlapped with body location, 0 otherwise). The second estimation of eye-gaze on the partner’s face, instead, relied on landmarks estimated using OpenPose. Specifically, we estimated the center of the face and the maximum extent of the face on the image. We then used this information to compute an ellipse around the center of the face. The results of this analysis indicated that individuals, on average, looked at the body of their partners 81.67% of the time, and specifically at the face 30.78% of the time. This confirms that participants were complying with the experimental instructions.

### Quantification and statistical analysis

#### Data analysis

Estimation of body movements from standalone video cameras relied on an automated machine learning-based estimation of body and hand landmarks (i.e., OpenPose^35^). We utilized a deep learning-based approach to extract 2-D body and hand landmarks from videos captured by the two video cameras. Every single frame from the videos was loaded and used as an input for a pre-trained multi-stage Convolutional Neural Network (CNN) that first jointly predicted a set of 2-D vector fields that encoded the location and orientation of limbs in the image domain – Part Affinity Fields (PAFs) – as well as confidence maps for body part detection. Next, body and hand landmark locations were predicted by a greedy inference that parsed the confidence maps and the PAFs. A collection of 25 body landmarks (across the head, torso, arms, and legs), as well as 42 hand landmarks were estimated (Figure 1B). A custom library (‘pytorch_openpose’) written in ‘opencv’ and ‘pytorch’ was used to load the pre-trained CNN model via OpenPose python API^35^ and predict 2-D body and hand landmarks for each frame in a video (see Figure 1B). For a fast prediction of the landmarks, an NVIDIA GeForce RTX 2060 SUPER graphics processing unit (GPU) was used.

Each landmark carried 2-D coordinates, i.e., movement on the x and y axes (relative to the camera’s field of view) over time. The landmark locations (in pixels) underwent a preprocessing procedure aimed at removing outlying values (3 standard deviations away from mean, computed within each trial, 0.68% of all data) or data points where the algorithm wasn’t able to predict body position (8.0% of all data) (Figure S5). The outlying values were then interpolated (1-D interpolation) and then the resulting time series were smoothed using a moving mean (window = 1 sec). We then computed a composite index of body part displacement over time (movement velocity) by first computing the Euclidean distance from the x and y coordinates, and then calculating the absolute value of the first derivative of the resulting time series (representing a rate of change of body part location, regardless of the specific direction). Next, the time series were smoothed using a (second) moving mean (window = 1 sec). The time series were further normalized (z-scoring) separately for each body part. Before combining the data associated with distinct landmarks, the data were visually inspected and trials associated with artifacts (i.e., high degree of variance) were removed (2.17%) (Figure S6). Finally, to reduce the dimensionality of the data, the 67 body landmarks were grouped to represent 10 different body parts: “Head” (mean of 4 landmarks), “Torso” (mean of 4 landmarks), “Left Arm” (mean of 3 landmarks), “Left Hand” (mean of 21 landmarks), “Right Arm” (mean of 3 landmarks), “Right Hand” (mean of 21 landmarks), “Left Knee” (1 landmark), “Right Knee” (1 landmark), ”Left Foot” (mean of 4 landmarks), and “Right Foot” (mean of 4 landmarks) as shown in Figure 1B.

#### Statistical analyses

We conducted two main analyses: the first aimed at comparing how much individual participants moved their body parts across distinct experimental conditions (i.e., irrespective of whether their partner moved simultaneously), and the second focused on interpersonal movement synchrony.

To approximate how much individual participants moved their body parts across conditions, we computed the range of movement velocities performed by each participant. We estimated this range (i.e., maximum – minimum value over time) from each body movement time series, separately for each body landmark, trial, and participant. The resulting values were averaged within each body part (based on the grouping described above) and across trials belonging to the same condition, as well as across participants belonging to the same dyad (the former was meant to ensure independence between the samples, which is an assumption of the parametrical tests used below). These data were entered into a 2×2×10 ANOVA with ‘interpersonal spatial proximity’, ‘visual contact’, and ‘body part’ as factors. We applied a Greenhouse–Geisser correction to the degrees of freedom in case there were any violations of sphericity. For the computation of effect sizes, we computed generalized eta-squared as it is a measure that is invariant across different research designs.^112^

Interpersonal movement synchrony was estimated by correlating the body movement time series of the two participants forming a dyad. For each 2-minute trial, Pearson’s correlation coefficients were calculated for every possible combination of participants’ body parts (e.g., P1-Head vs P2-Head, P1-Head vs P2-Left Arm, P1-Head vs P2-Torso, etc.), thus leading to a 10-by-10 correlation matrix for each of the 23 dyads and each of the 12 trials. Correlation matrices derived from the three trials associated to the same condition were subsequently averaged together to form one correlation matrix for each dyad and each condition. Furthermore, since the interaction between the participants was symmetrical (there were no assigned roles to any of the two participants), each correlation matrix was transformed into a (lower) triangular matrix by averaging across its main diagonal. For instance, the coefficients resulting from the correlation between the head of participant 1 and the torso of participant 2 were averaged with those resulting from the correlation between the torso of participant 1 and the head of participant 2.

We tested the significance of the correlation coefficients by first assessing their consistency across dyads (within each condition), and then by comparing experimental conditions using non-parametric permutation analyses.^113^ Testing the consistency of the correlation within each experimental condition, for each body part, we shuffled the time series data for one participant of the dyad (thereby destroying any relationship between the two time series due to time dependency between successive points of measurement). We then computed the (pseudo) correlation coefficient between the two time series. We performed this operation for all the dyads and computed a t-statistic comparing the coefficients vs. zero (indexing no correlation). This procedure was repeated 1000 times to generate a null distribution of (pseudo) t-values. The distribution of pseudo t-values was then compared to the genuine t-value obtained without shuffling. The percentage of cases where the pseudo data provided t-values higher than the genuine t-value corresponded to an estimate of the p value. Because this procedure was repeated for each body part, the resulting p values were corrected for multiple comparisons using a false discovery rate (FDR) correction.^114^ Condition-specific effect sizes (*Cohen’s d*) were calculated as their standardized mean difference.

Next, we compared the correlation coefficients across experimental conditions using a permutation test for a 2x2 ANOVA with ‘interpersonal spatial proximity’ and ‘visual contact’ as factors. For each body part, we shuffled the time series of one participant and computed the (pseudo) correlation coefficient, as for the previous analysis. We repeated this for all dyads and trials. We averaged the pseudo correlation coefficients across the trials to get 1 pseudo correlation coefficient for each condition and dyad. Further, we computed a pseudo F-value for the main effects of ‘interpersonal spatial proximity’ and ‘visual contact’ and the interaction based on these pseudo data. We repeated this process 1000 times to get null distributions of pseudo F-values. We then compared the genuine F-values to their corresponding null distributions of pseudo F-values. The percentage of cases where the pseudo data provided F-values higher than the genuine F-value corresponded to an estimate of the p value (for each of the main effects and interaction). The p values were similarly corrected for multiple comparisons using FDR correction.

Finally, we performed a cross-correlation analysis to understand whether the movements performed by one participant anticipated or followed the movements performed by his/her partner and, if so, with which time lag. Cross-correlation coefficients index the similarity between two time series as a function of their relative temporal displacement (i.e., given two vectors A and B, a cross-correlation coefficient indexes the similarity between A and time-shifted copies of B, at multiple time lags). To compute time lags in seconds, we interpolated the data time series for each participant, within each trial and body part, to obtain regular time points in the range 0–120 secs (in steps of 0.1 sec). We then computed cross-correlation coefficients (with ±10 secs time lags) within each trial, for all combinations of body parts. We then averaged the resulting coefficients across trials, focusing separately on the Vision and No Vision conditions, to form one vector of averaged coefficients (i.e., one coefficient per time lag). We further averaged these coefficients across dyads and then estimated the absolute lag associated with the highest correlation coefficient, separately for each body part combination.

## Data Availability

• Data are available via https://doi.org/10.48557/4K3WZI. • Code is available via https://github.com/ateshkoul/interpersonal_movement_synch/. • Any additional information required to reanalyze the data reported in this study is available from the lead contact upon request.
