# Supplementary material for: Interpersonal synchronization of spontaneously generated body movements
Source: iScience. 2023 Feb 1;26(3):106104. doi: 10.1016/j.isci.2023.106104 (PMC9958360; doi:10.1016/j.isci.2023.106104)
Supplement: Document S1. Figures S1–S6 [file mmc1.pdf]

iScience, Volume *26*

Supplemental information

Interpersonal synchronization of spontaneously generated body movements

Atesh Koul, Davide Ahmar, Gian Domenico Iannetti, and Giacomo Novembre

Interpersonal movement synchrony (all conditions – parametric analysis)
(linear correlations)

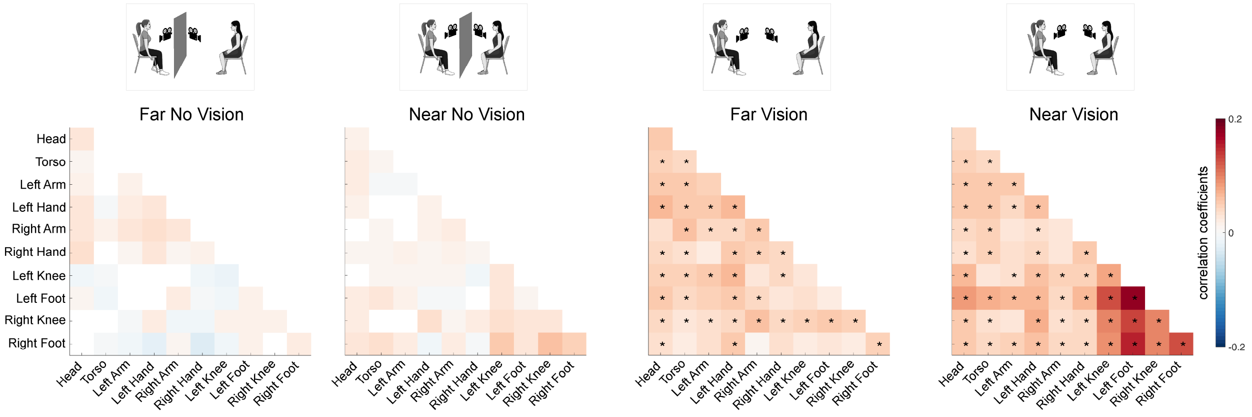

Figure S1. Interpersonal movement synchrony (all conditions – parametric analysis), related to Figure 3. We confirmed our interpersonal movement synchrony results (Figure 3) using a parametric analysis. T-tests were performed contrasting the correlation coefficients vs. zero (indexing no correlation). The resulting p-values were corrected for multiple comparisons using FDR correction. Only the Vision conditions were associated with significant correlations (*p < 0.05 FDR-corrected).

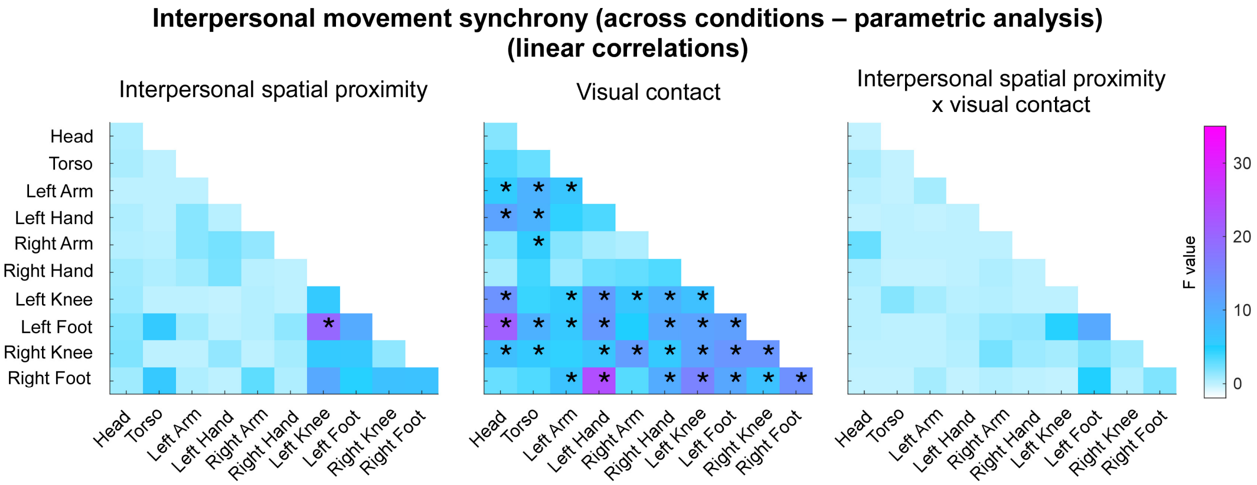

Figure S2. Effects of interpersonal spatial proximity and visual contact on interpersonal synchrony of spontaneous movements (across conditions – parametric analysis), related to Figure 4. Using parametric ANOVAs, we replicated the results reported in Figure 4. The results are corrected for multiple comparisons using FDR correction (*$p < 0.05$ FDR-corrected).

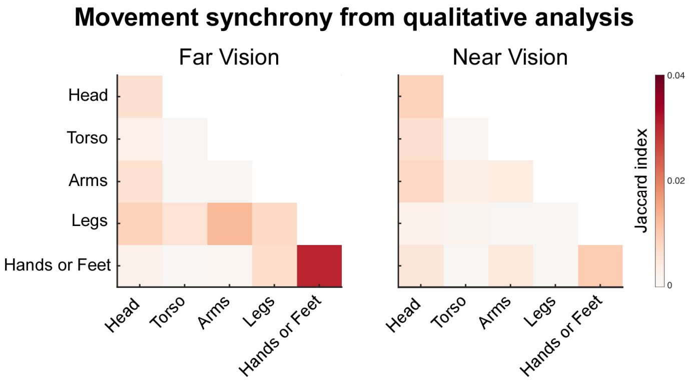

Figure S3. Interpersonal movement synchrony from qualitative analysis, related to Figure 3. In addition to the quantitative analysis reported in the manuscript, we also performed a qualitative analysis of body movement to understand whether spontaneous interpersonal movement synchrony could be evaluated by a human observer. A human rater observed the video recordings obtained from the head-mounted camera of the eye-tracker and manually coded perceived spontaneous movements of multiple body parts in each participant in a frame-by-frame manner. The manual coding was based on a predefined list of movements of interest. The coding was accomplished using MATLAB's video labeler toolbox (MATLAB, Computer Vision Toolbox, 2020b). In accordance with previous manual coding procedures (e.g.,[1–3]) participants' movements were classified according to an anatomical basis. Movements of the head and neck were labelled as "Head", movements of the thorax or abdomen as "Torso", movements of the entire left and right arms as "Arms", and movements of the left and right legs as "Legs". Moreover, small movements in the left and right hands or feet, which did not involve the whole arms or legs (e.g., fidgeting), were labelled as "Hands or Feet". Since the manually labelled time series data were binary (i.e., each datapoint indexes the presence or absence of movement), we used the Jaccard index (or Jaccard similarity coefficient[4]) to quantify the similarity (or match) between the time series of the two participants forming a dyad [ranging between 0 (no match) to 1 (perfect match)]. The Jaccard index was computed for each body part combination and for each trial. The indices were then averaged across the trials to get one Jaccard index for each body part combination, condition, and dyad. We tested the significance of the Jaccard indices by assessing their consistency across dyads, separately for Far Vision and Near Vision conditions, using one-sample t-tests vs. zero (indexing no match). This analysis revealed, for the Far Vision condition, a slightly significant (at uncorrected p-values) synchronization between body parts such as "Hands or Feet" and "Head" ($p = 0.021$ uncorrected), as well as between "Hands or Feet" and "Hands or Feet" ($p = 0.037$ uncorrected). Similarly, for the Near Vision condition, a trend towards significance was found (at uncorrected p-values) between body parts such as "Head" and "Head" ($p = 0.034$ uncorrected), "Hands or Feet" and "Head" ($p = 0.045$ uncorrected), and "Hands or Feet" and "Arms" ($p = 0.048$ uncorrected). Although none of these results survived FDR correction, they are generally consistent with what was yielded by the quantitative analysis reported in the manuscript (Figure 3). Furthermore, the results suggest that the described interpersonal synchrony might be partially detected by a human observer – although not as precisely as by the algorithm used for the quantitative analysis. This deserves to be thoroughly investigated in the future.

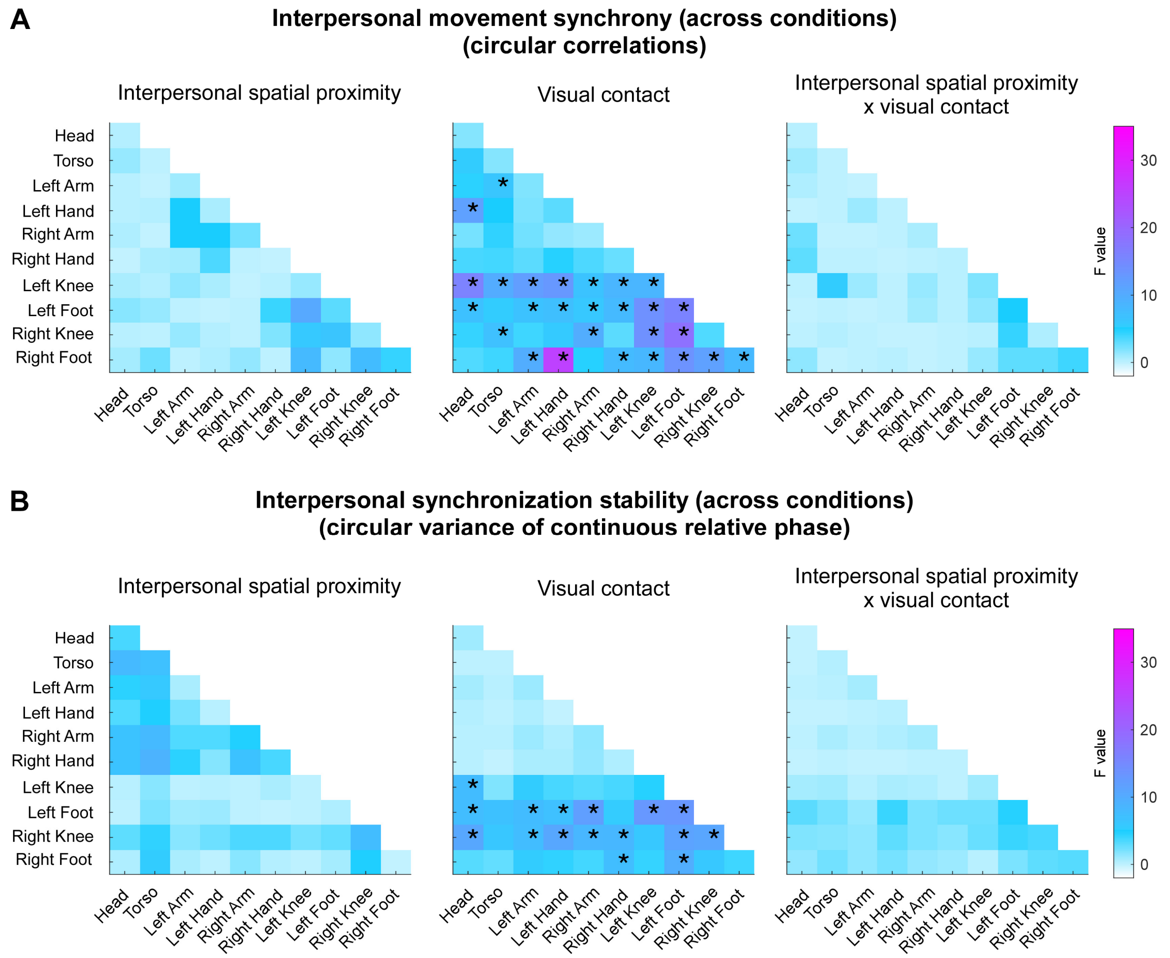

Figure S4. Effects of interpersonal spatial proximity and visual contact on interpersonal synchrony of spontaneous movements (across conditions - circular measures), related to Figure 4. We replicated the results reported in Figure 4 using two circular measures of IMS. The figure depicts the F-values resulting from parametric ANOVAs on such circular measures (described below), comparing all experimental conditions (separately for each body part combination). A: We first computed the circular correlation of the two movement time series. Circular correlation is a parallel of Pearson's product moment correlation for circular data[5]. The phase angles were computed from the movement time series by centering their range around zero, transforming them into analytic signals using Hilbert transform, and then extracting the instantaneous phase angles[6,7]. Circular correlations were obtained from these phase time series using the function "circ_corrcc" from the "Circular Statistics Toolbox"[8]. We performed ANOVAs comparing circular correlation coefficients across conditions, separately for each body part combination, and corrected the resulting p-values for multiple comparisons using FDR correction. The ANOVAs yielded several significant main effects of "visual contact" on interpersonal movement synchrony, entailing multiple body part combinations. The main effect of "interpersonal spatial proximity" and the interaction between "interpersonal spatial proximity" and "visual contact" were not statistically significant for any body part combination (*$p < 0.05$ FDR-corrected). B: same as (A) but with a measure of interpersonal synchronization stability: circular variance of the continuous relative phase. Circular variance indexes the proportion of relative phase relationships that are visited by the two movement time series[9]. We used the procedure outlined above for estimating phase angles. We then computed the relative phase angle between the two phase time series[7]. Circular variance was computed using the function "circ_var" from the "Circular Statistics Toolbox"[8]. We performed ANOVAs to compare variance across conditions, separately for each body part combination, and corrected the p-values for multiple comparisons using FDR correction. Only main effects of "visual contact" were statistically significant, and this was so for multiple body part combinations (*$p < 0.05$ FDR-corrected).

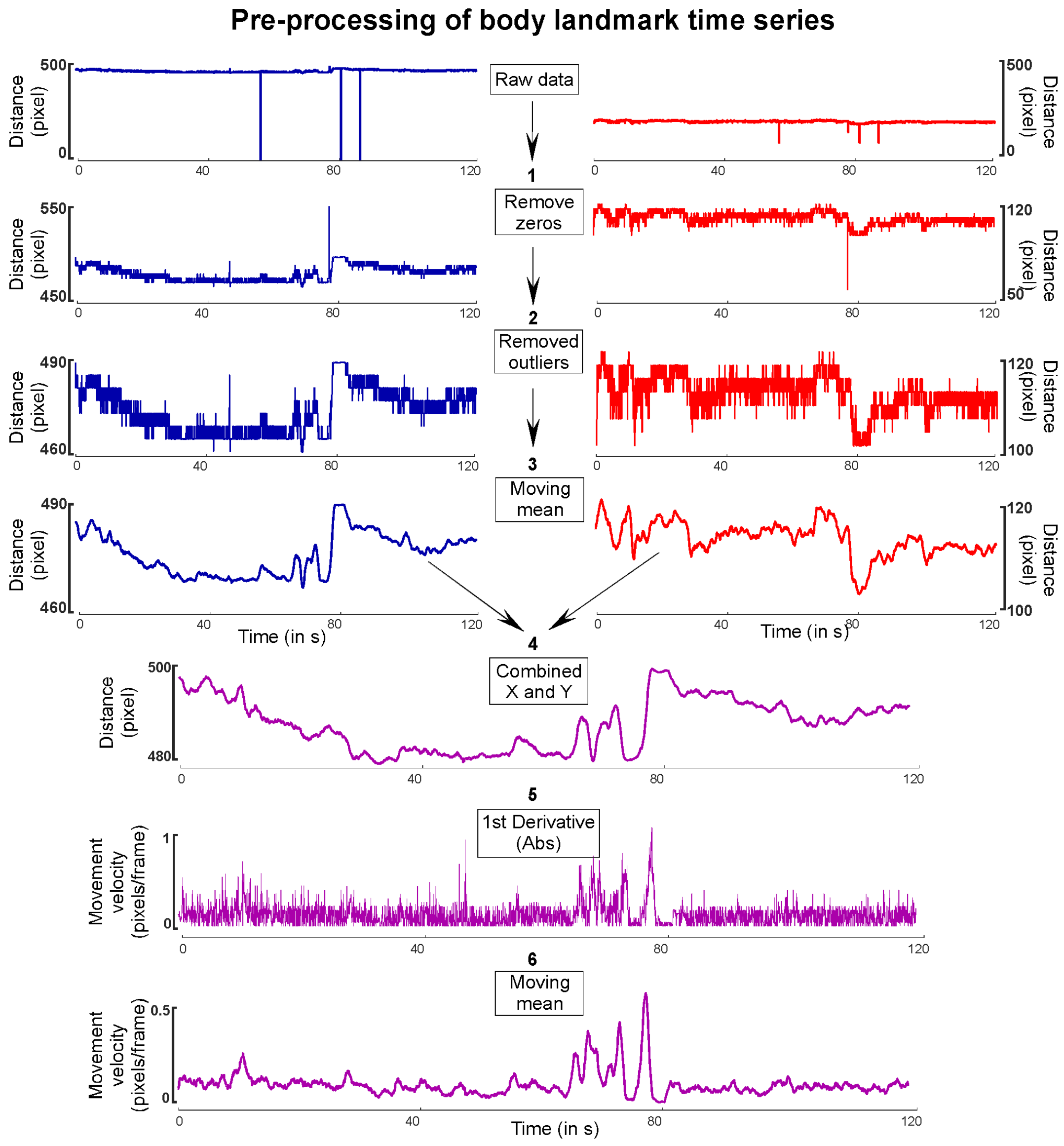

Figure S5. Preprocessing of body landmark time series, related to STAR Methods. Visual representation of the preprocessing procedure for the computation of movement velocity. The preprocessing procedure first involved the removal of data points where the algorithm wasn't able to predict body position (zero values) (step 1 in the figure), and outliers (defined as values exceeding 3 standard deviations from the mean of each trial) (step 2). The missing data points in the time series were subsequently interpolated using MATLAB's 1-D interpolation (table lookup). The data were then smoothed using a moving mean (window = 1 sec) (step 3), after which the Euclidean distance of a body landmark from the x and y coordinates was computed (step 4). To compute an estimate of body movement, we used the absolute value of the first derivative (step 5). This transformation served to identify body part displacement over time, irrespective of the spatial direction of the movement (similar to computing movement speed). Finally, a second moving mean of 1 sec (step 6) was performed on the resulting movement time series.

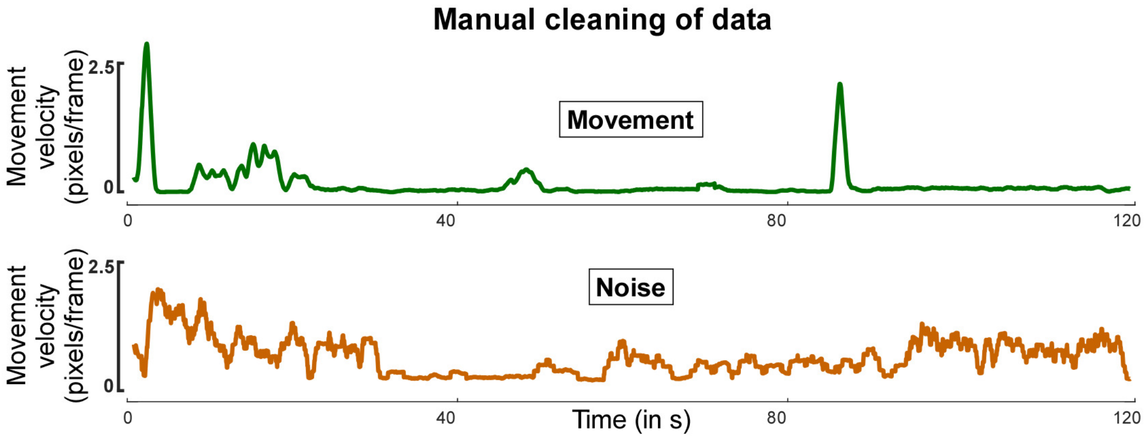

Figure S6. Manual verification of data cleaning procedure, related to STAR Methods. The preprocessed body landmark time series were further visually inspected for noise. Each time series was visually compared with its corresponding video recording (body part by body part) to detect any artifacts (e.g., high degree of variance). Such trials were removed from the further data analysis (2.17% of all data). Example time series for typical (upper panel) and noisy body landmarks (lower panel) are shown in the figure.

SI References:

1. Bernieri, F.J., Reznick, J.S., and Rosenthal, R. (1988). Synchrony, pseudosynchrony, and dissynchrony: Measuring the entrainment process in mother-infant interactions. Journal of Personality and Social Psychology *54*, 243–253. 10.1037/0022-3514.54.2.243.

2. Condon, W.S., and Ogston, W.D. (1966). Sound film analysis of normal and pathological behavior patterns. The Journal of Nervous and Mental Disease *143*, 338–347. 10.1097/00005053-196610000-00005.

3. Kendon, A. (1970). Movement coordination in social interaction: Some examples described. Acta Psychologica *32*, 101–125. 10.1016/0001-6918(70)90094-6.

4. Kelleher, J.D., Namee, B.M., and D'Arcy, A. (2015). Fundamentals of Machine Learning for Predictive Data Analytics (The MIT Press).

5. Jammalamadaka, S. Rao and SenGupta, A. (2001). Topics in Circular Statistics 5th ed. (world scientific).

6. Lamb, P.F., and Stöckl, M. (2014). On the use of continuous relative phase: Review of current approaches and outline for a new standard. Clinical Biomechanics *29*, 484–493. 10.1016/j.clinbiomech.2014.03.008.

7. Pikovsky, A., Kurths, J., Rosenblum, M., and Kurths, J. (2001). Synchronization: A Universal Concept in Nonlinear Sciences (Cambridge University Press).

8. Berens, P. (2009). CircStat : A *MATLAB* Toolbox for Circular Statistics. J. Stat. Soft. *31*. 10.18637/jss.v031.i10.

9. Schmidt, R.C., and Fitzpatrick, P. (2019). Embodied Synchronization and Complexity in a Verbal Interaction. Nonlinear dynamics, psychology, and life sciences *23*, 199–228.
